# Caspase-independent apoptosis in infected macrophages triggered by sulforaphane via Nrf2/p38 signaling pathways

**DOI:** 10.1038/cddiscovery.2015.22

**Published:** 2015-08-24

**Authors:** M Bonay, A-L Roux, J Floquet, Y Retory, J-L Herrmann, F Lofaso, TB Deramaudt

**Affiliations:** 1 Laboratoire de Physiologie TITAN, INSERM U1179, UFR des Sciences de la Santé—Simone Veil, Université de Versailles Saint-Quentin-en-Yvelines, Montigny-le-Bretonneux, France; 2 Service de Physiologie-Explorations Fonctionnelles, Hôpital Ambroise Paré, Assistance Publique-Hôpitaux de Paris, Boulogne, France; 3 LIA-BAHN (Laboratoire International Associé—Biologie Appliquée Handicap Neuromusculaire), CSM (Centre Scientifique de Monaco), Monaco; 4 INSERM U1173, Equipe EPIM, UFR des Sciences de la Santé—Simone Veil, Université de Versailles Saint-Quentin-en-Yvelines, Montigny-le-Bretonneux, France

## Abstract

*Mycobacterium abscessus* (*Mabs*), a non-tuberculous mycobacterium, is an emerging and rapidly growing opportunistic pathogen that is frequently found in patients with cystic fibrosis and in immunosuppressed patients. Its high tolerance to antibiotics is of great concern for public health. In this study, our results showed that human THP-1-derived macrophages infected with *M. abscessus* presented an increase in ROS production and cell necrosis. In addition, *M. abscessus* infection triggered activation of the Nuclear factor E2-related factor 2 (Nrf2) signaling pathway, and the induction of HO-1 and NQO1 expression levels. Interestingly, pretreatment of macrophages with sulforaphane (SFN), an activator of the antioxidant key regulator Nrf2, followed by *M. abscessus* infection significantly decreased mycobacterial burden. We demonstrated that this reduction in mycobacterial growth was due to an activation in cell apoptosis in SFN-pretreated and *M. abscessus*-infected macrophages. Pretreatment with specific MAPK inhibitors, PD98059, SP600125, and SB203580 to ERK, JNK, and p38 respectively, failed to inhibit induction of Nrf2 expression, suggesting that Nrf2 signaling pathway was upstream of MAPK signaling. Activation of cell apoptosis was caspase 3/7 independent but p38 MAPK dependent. Moreover, p38 MAPK induction was abolished in macrophages transfected with Nrf2 siRNA. In addition, p38 inhibitor abolished Nrf2-dependent apoptosis in infected macrophages. Taken together, our results indicate that modulation of the Nrf2 signaling using Nrf2 activators may help potentiate the actual drug therapies used to treat mycobacterial infection.

## Introduction

Mycobacteria included in the group of rapid growing mycobacteria (RGM) are increasingly becoming a public health concern worldwide. RGM have proven to be a great challenge to eradicate since these organisms present a high tolerance to antibiotics, which greatly limits treatments, and are highly prevalent in immunosuppressed patients. *Mycobacterium abscessus* (*Mabs*), a non-tuberculous mycobacterium of the RGM group, is an opportunistic pathogen. *M. abscessus* is an emerging pathogen that has been increasingly involved in patients with cystic fibrosis and in immunosuppressed patients,^1–3^ and more generally in exacerbation of lung infections. Association of *M. abscessus* with patients suffering from a pre-existing condition and at risk of developing chronic airway infections makes for a poor clinical outcome. Alike *M. tuberculosis*, *M. abscessus* survives in phagocytic cells and uses the host immune cells as a reservoir for proliferation. Recent *in vitro* studies on THP-1-derived macrophages showed that *M. abscessus* appears to thrive in oxidative environment. The bacterial growth is enhanced in oxidative condition such as the presence of oxygen-free radicals, while its growth is inhibited in the presence of oxidant scavengers such as MnTE-2-PyP and N-acetyl-L-cysteine.^4^ MnTE-2-PyP is able to diminish *M. abscessus* load in infected macrophages by inducing the fusion of mycobacteria-containing phagosomes with lysosomes into phagolysosomes, thus promoting cell survival.^5^

This imbalance between oxidants and antioxidants in the infected host cell activates the antioxidant signaling pathway controlled by the transcriptional factor Nuclear factor E2-related factor 2 (Nrf2). Nrf2 is a key regulator in adaptive responses to oxidative stress by inducing the transcription of antioxidant and cytoprotective genes.^6^ In normal physiological conditions, NRF2 is sequestered in the cytoplasm by its negative regulator Kelch-like ECH-associated protein 1 (Keap1). The Nrf2/Keap1 complex is rapidly led to ubiquitin degradation via the Keap1-Cullin-3 based E3 ligase complex.^7^ Upon oxidative stress, infection, or chemical stimulation, Nrf2 is released from Keap-1, and translocates to the nucleus, where it heterodimerizes with transcription factors including Maf, c-Jun, c-Fos, and members of the AP-1 family.^8^ The cofactor complex binds specifically to the antioxidant responsive element (ARE) sequences found in a wide range of antioxidant genes coding for antioxidant enzymes such as NADPH quinone oxidoreductase-1, epoxide hydrolase-1, HO-1, UDP-glucuronyl transferase, and glutathione-S-transferases.^9,10^

Previous studies have shown that *M. tuberculosis* at high MOI escapes bactericidal killing in infected host cells by eliciting necrosis cell death.^11^ Activation of this energy-independent cell death by *M. tuberculosis* allows the release of mycobacteria and subsequent infection of neighboring phagocytes. An important defense mechanism utilized by the innate immune system is the triggering of the programmed cell death, also known as cell apoptosis, to reduce the viability of pathogens. Cell apoptosis is an energy-dependent process and presents bactericidal properties. Apoptotic bodies issued from infected apoptotic macrophages maintain plasma membrane integrity, and thus enable antigen presentation, which facilitate T-cell response and induce direct mycobacterial killing by uninfected neighboring macrophages.^12^

Since very little is known about the mechanism by which *M. abscessus* infects and disseminates in phagocytic cells, we used human THP-1-derived macrophages to explore the intracellular processes involved in *M. abscessus* infection. Moreover, we investigated the role of Nrf2 and its antioxidant signaling pathway against *M. abscessus* infection of THP-1-derived macrophages. We showed that pretreatment of macrophages with an activator of Nrf2, sulforaphane (SFN), before *M. abscessus* challenge decreased mycobacterial growth by activating a caspase-independent apoptotic response. Activation by Nrf2 of this programmed cell death in infected macrophages was p38 MAPK dependent. Our results suggest that Nrf2 activators may be used as therapeutic treatments in addition to the actual multi-drug therapies used in patients infected with *M. abscessus*.

## Results

### Activation of the Nrf2-dependent antioxidant pathway by SFN and/or *M. abscessus*

**Figure 1:**
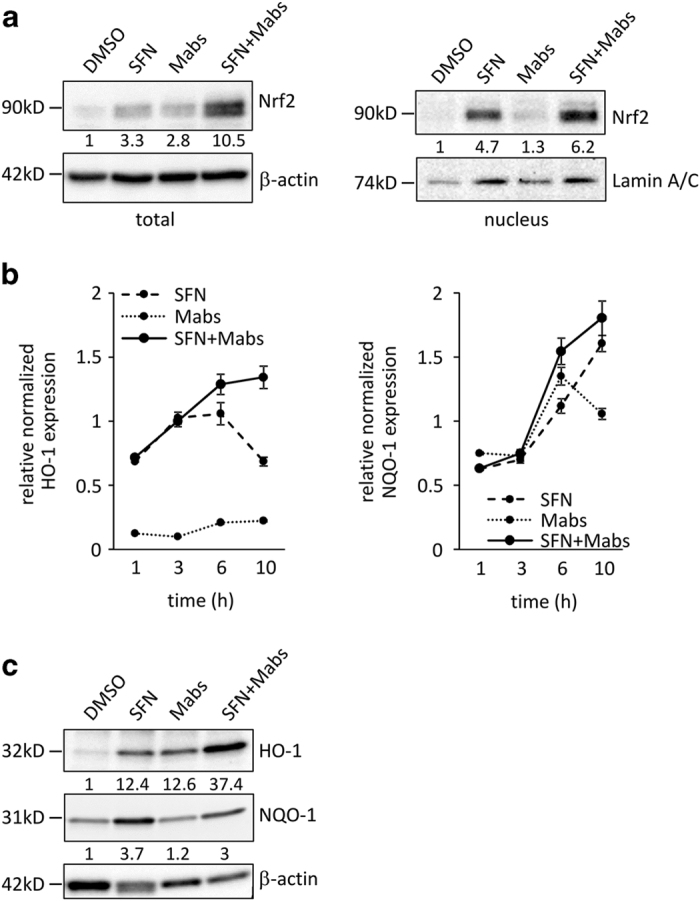
(**a**) *Mabs* induces Nrf2 protein expression level in THP-1-derived macrophages. Protein expression levels were normalized to *β*-actin in the total protein extracts, and to Lamin A/C in the nuclear extracts. (**b**) RT-qPCR: Nrf2 signaling pathway is activated by *M. abscessus* and SFN. The Nrf2 targets HO-1 and NQO1 mRNA expression levels were normalized to the housekeeping gene ubiquitin. Data shown are the means±S.E.M. of three independent experiments done in triplicates. (**c**) Protein expression levels of HO-1 and NQO1 were detected by immunoblotting. Densitometric quantification of protein signals was normalized to *β*-actin.

To determine the effect of *Mabs* infection on the antioxidant pathway, we sought to define the expression level of Nrf2 in macrophages derived from phorbol 12-myristate 13-acetate (PMA)-differentiated human THP-1 cells. Macrophages were pretreated 3 h with SFN before infection with *Mabs*. *Mabs* infection induced Nrf2 protein expression level 24 h post infection 2.8-fold higher as compared with that of DMSO (Figure 1a). Nrf2 expression level in macrophages treated with SFN was increased more than 3-fold compared with DMSO-treated cells. Interestingly, *Mabs* infection in SFN-treated macrophages strongly increased Nrf2 protein level to 10.5-fold. Nrf2 activation is confirmed by analyzing nuclear proteins extracted from SFN-pretreated and/or *Mabs-*infected macrophages. Immunoblots revealed that Nrf2 is translocated to the nucleus in SFN-pretreated macrophages (4.7-fold increase) and confirmed the strong increase in Nrf2 previously seen in total protein extracts in SFN-pretreated/*Mabs-*infected cells (6.2-fold increase). Interestingly, infection with *Mabs* alone augmented Nrf2 in the total protein extracts but is not reflected in the nuclear Nrf2.

Quantitative PCR of two downstream targets of Nrf2 showed a rapid and significant increase in mRNA levels of heme oxygenase-1 (HO-1) and NAD(P)H dehydrogenase, quinone 1 (NQO1) with the persistence of the mRNA levels 10 h after SFN treatment alone or SFN pretreatment/*M. abscessus* infection of THP-1-derived macrophages (Figure 1b). Infection with *Mabs* alone led to a weak induction of HO-1 mRNA or an induction of NQO1 mRNA that decreased 6 h after *Mabs* infection. Immunoblots using total protein extracts of macrophages lysed 24 h post infection confirmed the significant 37.4-fold increase in HO-1 protein level in SFN-pretreated/*Mabs-*infected cells (Figure 1c) comparable to the pattern observed with total Nrf2 extracts. It is noteworthy that NQO1 protein levels showed a comparable pattern to the one seen in nuclear Nrf2 immunoblot.

### SFN-treated cells showed decreased mycobacterial proliferation

**Figure 2:**
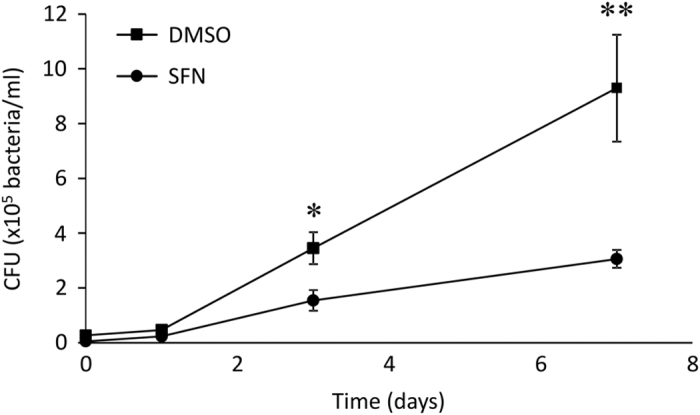
SFN showed a decrease in mycobacterial burden in infected macrophages. THP-1-derived macrophages were pretreated with DMSO or 10 *μ*M SFN 3 h before infection with *M. abscessus* for 3 h. The unincorporated mycobacteria were incubated in culture medium containing 250 *μ*g/ml amikacin for 1 h before thorough washes. The cells were then incubated in medium with 50 *μ*g/ml amikacin for the indicated time period (0, 1, 3, and 7 days). Intracellular mycobacteria were released by cell lysing in ice-cold water, serially diluted, and seeded on agar plates. Colony-forming units were counted 5 days after incubation at 37 °C. The graph represents the means±S.E.M. of two independent experiments done in triplicates. *P<0.03, **P<0.01.

Since *M. abscessus* has been previously shown to proliferate more favorably in oxidative stress conditions,^5^ we sought to determine the effect of Nrf2 activation on the bactericidal activity of macrophages against *M. abscessus*. THP-1-derived macrophages were infected with *Mabs* for 3 h at a multiplicity of infection of 10 bacilli for 1 cell, thoroughly washed and incubated in amikacin and SFN supplemented medium. After the indicated post infection times, macrophages were lysed and live internalized mycobacteria were quantified using the colony counting method (colony-forming unit, CFU). The results showed an increase in *Mabs* viability and proliferation over the 7- day observation period in DMSO-pretreated cells (Figure 2). Interestingly, SFN-pretreated macrophages infected with *Mabs* yielded a 2-fold decrease in CFU at day 3 post infection and a 3-fold decrease at day 7 post infection compared with DMSO-treated cells. These data suggest that SFN may have an inhibitory effect on mycobacterial proliferation in macrophages.

### SFN-induced mycobacterial growth decrease is independent from phagosomal acidification

**Figure 3:**
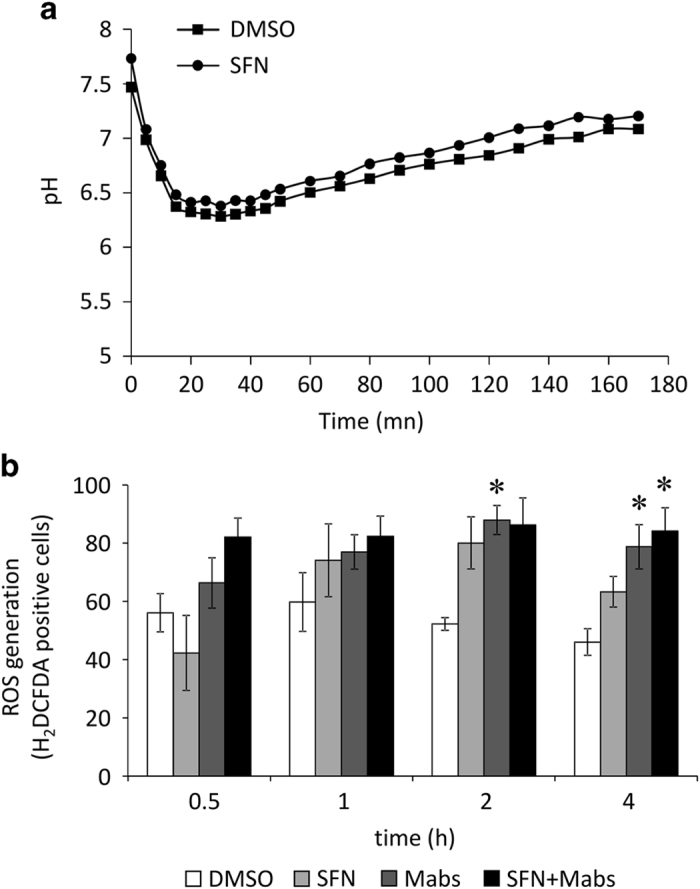
(**a**) SFN does not interfere with phagosomal maturation. The pH acidification was determined by quantifying FITC signal intensities, which is pH dependent in THP-1 infected with FITC-labeled *Mabs*-mCherry. The pH-independent mCherry fluorochrome was used as an internal control to estimate the amount of labeled bacteria per well. Graph shows one representative experiment out of ten. Data represent the means±S.E.M. of 10 independent experiments done in triplicates. (**b**) ROS response in *M. abscessus-*infected macrophages. THP-1-derived cells were infected and ROS was detected using H_2_DCFDA fluorescence labeling. Data acquisition (*n*=5000 events) and analysis were performed using the MARKII imaging flow cytometer. The graph represents the means±S.E.M. of three independent experiments. *P<0.05 compared with DMSO-treated cells.

Numerous pathogens including *M. tuberculosis* have developed complex mechanisms to survive and proliferate in host phagocytes by interfering with the phagosomal maturation process thus blocking the fusion of phagosomes with lysosomes and the generation of phagolysosomes. This impediment prevents exposition of the ingested bacteria to reactive oxygen metabolites, lysosomal hydrolases, and general acidification of the phagolysosome to pH below 5.0, allowing the use of macrophages as proliferation reservoir.^13^ To determine whether *Mabs* or/and SFN has an effect on phagosomal maturation, phagosome acidification assay was used. *Mabs* expressing mCherry fluorochrome was surface labeled with the pH-sensitive fluorescein-5-isothiocyanate (FITC) and were used to infect THP-1-derived macrophages. FITC, which emission intensities are pH dependent, was used to determine phagosomal acidification, while mCherry, a pH-independent fluorochrome, was used as an internal indicator of the number of bacteria (Figure 3a). Our results showed that *Mabs* efficiently inhibited phagosomal acidification in DMSO treated macrophages (pH=6.27±0.07). Interestingly, reduction in mycobacterial burden in SFN-treated macrophages (Figure 2) was not due to a decrease in phagosomal pH since the phagosomes of SFN-treated macrophages remained stable at pH 6.17±0.14 (Figure 3a).

Using the ROS-sensitive indicator, H_2_DCFDA, we analyzed the intracellular oxidative stress level in *Mabs-*infected macrophages pretreated with SFN or DMSO (Figure 3b). ROS production was significantly augmented by *Mabs* infection at 2 h post infection and in SFN-pretreated/*Mabs*-infected macrophages compared with DMSO-treated cells. Macrophages pretreated with SFN alone did not show a significant modulation of the ROS-sensitive indicator compared with DMSO-treated cells. These data suggest that infection with *Mabs* increases ROS production with no effect of SFN pretreatment on the latter.

### *Mabs* infection induces cell necrosis in THP-1-derived macrophages

**Figure 4:**
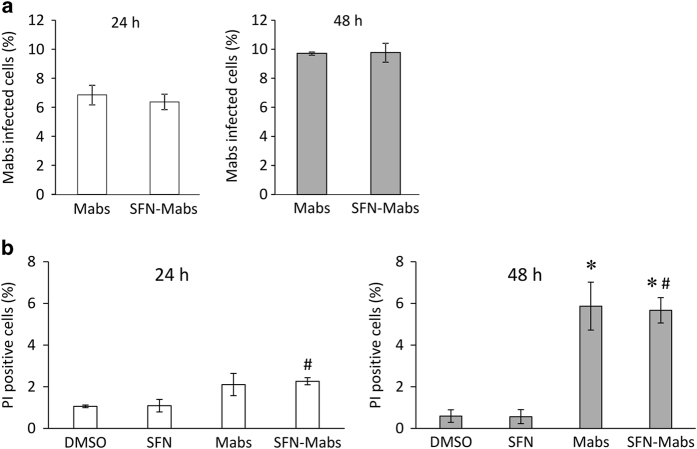
SFN does not modulate mycobacterial intake by THP-1 cells. (**a**) THP-1 infected with fluorescent *M. abscessus*. The graphs represent the means±S.E.M. of two independent experiments of 10 000 events each acquired by MARKII imaging flow cytometer. (**b**) Cell necrosis was determined using propidium iodide (PI) in THP-1 infected with *Mabs*. The graphs at 24 and 48 h represent the means±S.E.M. of two independent experiments of 10 000 events each. *P<0.02 compared with DMSO-treated cells, and ^#^ *P*<0.02 compared with SFN-treated cells.

*Mabs* expressing mCherry was used to infect for 3 h THP-1-derived macrophages that were pretreated with SFN or DMSO. Analysis by imaging flow cytometry on 24 h and 48 h post-infected cells showed first an increase in the number of bacteria infected cells at 48 h post infection compared with that of 24 h post infection cells, and second, that SFN pretreatment had no significant effect on *Mabs* phagocytosis by THP-1-derived macrophages (Figure 4a).

Unlike the well-studied *M. tuberculosis*, very little is known about the effect of *Mabs* infection and cell death. To establish the effect of *M. abscessus* infection on cell necrosis, THP-1-derived macrophages were infected with *Mabs*-mCherry for 3 h and incubated for 24 and 48 h. Cells were then stained with propidium iodide (PI) and analysis was performed by imaging flow cytometry. At 24 h post infection, SFN alone did not significantly induce cell necrosis, nor did *M. abscessus* infection alone or the combination of both. Interestingly, *M. abscessus*-dependent cell necrosis was raised from 2.26±0.17% at 24 h post infection to 5.66±0.6% at 48 h after infection (Figure 4b). Similar results were obtained in SFN-pretreated and *Mabs-*infected THP-1, suggesting that SFN pretreatment did not modulate *M. abscessus-*induced cell necrosis.

### *Mabs* infection induces apoptosis in SFN-treated THP-1-derived macrophages

**Figure 5:**
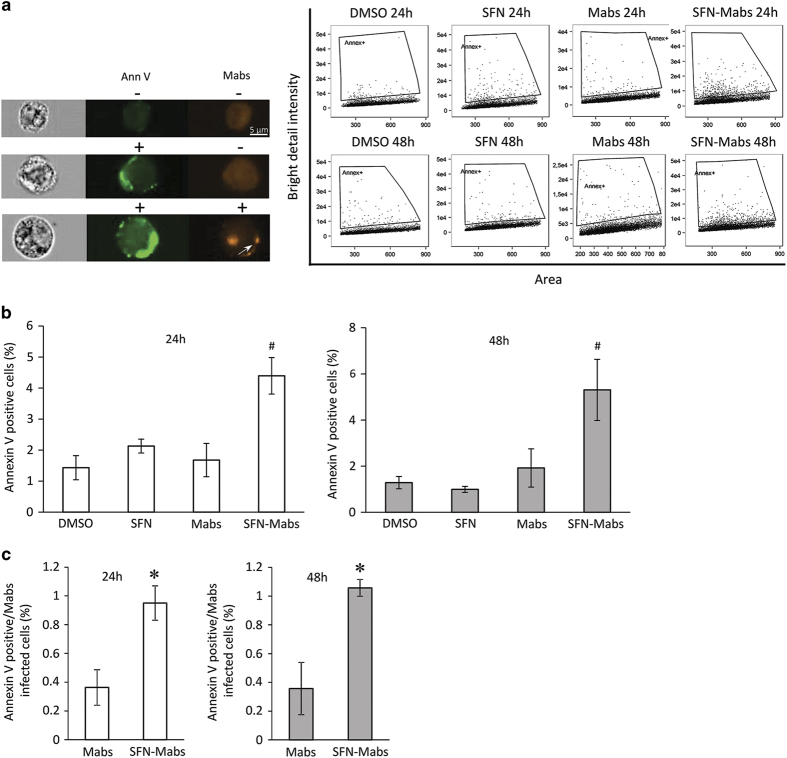
Apoptosis induction by SFN in *M. abscessus*-infected macrophages. THP-1-derived macrophages pretreated with SFN or DMSO for 3 h were infected with *M. abscessus* at the MOI of 10:1 for 24 or 48 h. (**a**) Annexin V-FITC (green) and *Mabs*-mCherry (red; white arrow) were excited by 488 and 568 nm lasers, respectively, and each individual macrophage was imaged by the 40× objective of the ImageStream MARKII cytometer and data analysis was performed (*n*=10 000 cells). (**b**) The percentage of apoptotic cells was determined after the indicated post infection times using Annexin V-FITC labeling. ^#^ *P*<0.03 compared with SFN-treated cells. (**c**) Colocalization of *Mabs*-mCherry in annexin V-FITC positive cells. Data represent the means±S.E.M. of three independent experiments. *P<0.04 compared with *Mabs*-mCherry infected cells.

Since SFN did not affect phagosomal acidification nor cell necrosis, we hypothesized that SFN might reduce mycobacterial burden through cell apoptosis. Indeed, one of the mechanisms phagocytes utilize to increase mycobactericidal activity is for the infected macrophages to trigger apoptosis, generating apoptotic bodies that will induce killing by non-infected bystander macrophages.^14^ To test the apoptotic response of macrophages following mycobacterial infection, THP-1-derived macrophages were infected with *Mabs* and left incubated for 24 and 48 h. Early cell apoptosis was detected using an Annexin V-FITC probe and quantified by imaging flow cytometry (Figure 5a).

As can be seen, no significant difference in cell apoptosis between *Mabs*-infected macrophages at 24 or 48 h and DMSO or SFN alone treated cells (Figure 5b). Interestingly, macrophages that were pretreated with SFN and subsequently infected with *Mabs* showed an increase in cell apoptosis at 24 and 48 h post infection (Figure 5b). Quantification of apoptotic cells that colocalized with mycobacteria showed a significant increase in SFN-pretreated cells compared with *Mabs*-infected cells (Figure 5c), thus suggesting a role of SFN in inducing cell apoptosis in macrophages infected by *Mabs*.

### *Mabs* infection in SFN-treated cells induces cell apoptosis in a caspases 3/7 independent pathway

**Figure 6:**
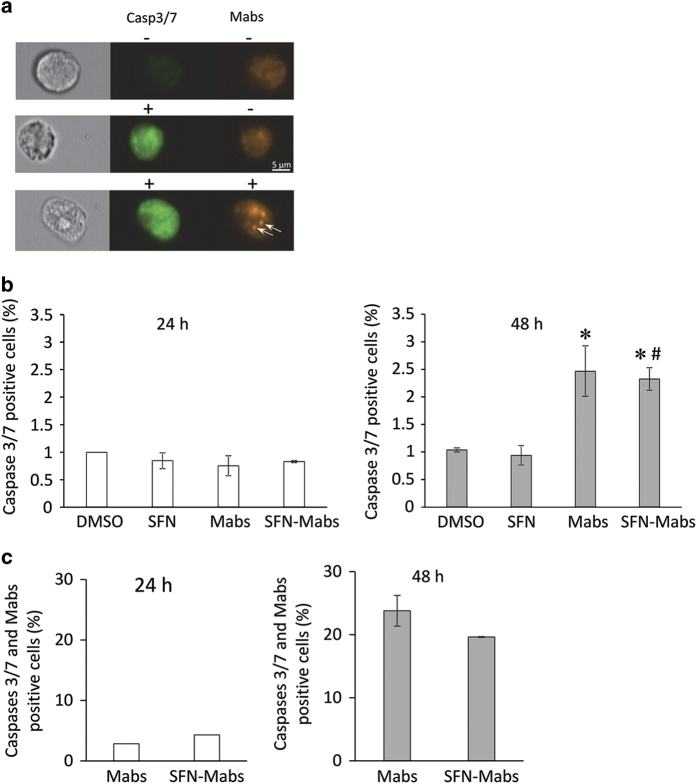
Macrophage apoptosis induction by SFN is caspase 3/7 independent. THP-1-derived macrophages pretreated with SFN or DMSO were pretreated for 3 h before mycobacterial infection. Caspase 3/7 activities were determined using FLICA inhibitor probe. (**a**) FLICA inhibitor probe (green) and *Mabs*-mCherry (red indicated by white arrows) were excited by a 488-nm laser and a 568-nm laser, respectively. Each individual macrophage was imaged by the 40× objective of the ImageStream MARKII cytometer and data analysis was performed on *n*=10 000 events. (**b**) Results were expressed as the percentage of FLICA-positive cells. *P<0.04 compared with DMSO-treated cells, and ^#^ *P*<0.001 compared with SFN-treated cells. (**c**) Colocalization of *Mabs*-mCherry in FLICA-positive cells. Data represent the means±S.E.M. of three independent experiments.

Since activation of the caspase cascade is an essential process in cell apoptosis,^15^ we sought to determine whether the cell apoptosis observed in SFN-treated and *Mabs-*infected macrophages was caspase dependent. THP-1 cells treated with SFN were infected with *Mabs*, and activated caspases 3/7 were assessed using a non-cytotoxic fluorescent inhibitor of caspases (FLICA) probe that binds covalently to active caspases 3 and 7 and analyzed by imaging flow cytometry (Figure 6a). At 24 h post infection, no significant activation of caspases 3/7 was seen in SFN-pretreated, *Mabs-*infected, and SFN-pretreated–*Mabs*-infected macrophages compared with DMSO-treated cells (Figure 6b). However, cells incubated 48 h after infection showed a significant increase in caspases 3/7 activation in *Mabs*-infected cells compared with DMSO-treated cells and in SFN-pretreated–*Mabs*-infected macrophages compared with SFN-pretreated cells. Treatment with SFN did not modify caspases 3/7 activity in the presence or absence of *Mabs*. Colocalization of caspases 3/7 positive cells and *Mabs*-mCherry positive cells confirmed that SFN does not activate caspases 3/7 pathway in infected cells compared with untreated cells (Figure 6c).

### Nrf2 regulates the MAPK signaling pathway

**Figure 7:**
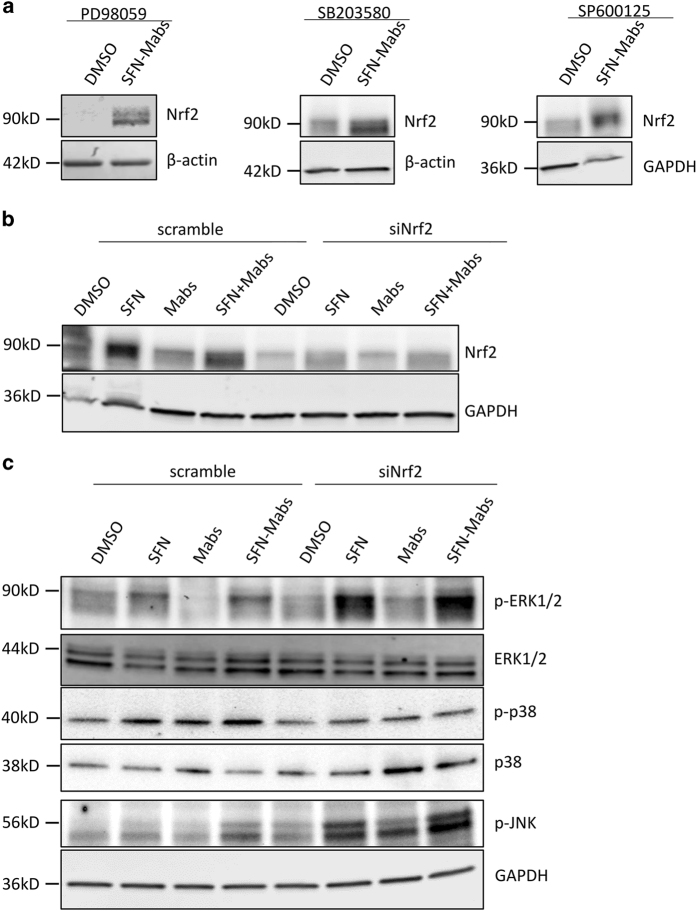
Nrf2 signaling pathway regulates MAPK cascade. (**a**) THP-1-derived macrophages were pretreated with MAPK-specific inhibitors PD98059, SB203580, and SP600125 of ERK, p38, and JNK, respectively, 2 h before SFN stimulation and/or *Mabs* infection. Cells were lysed 24 h after infection and protein lysates were analyzed by western blot. (**b**) THP-1-derived macrophages were transfected with scrambled or Nrf2 siRNA 24 h before SFN stimulation and/or *M. abscessus* infection. Cells were incubated for an additional 24 h after infection before lysis and western blots were performed using Nrf2 antibody and GAPDH antibody, as an internal control. (**c**) Immunoblots were performed on total protein lysates using specific phosphorylated antibodies against ERK, JNK, and p38.

MAPK signaling pathway is known to have an important role in the regulation of cell death decisions, which prompted us to hypothesize that MAPK cascade may be involved in the caspase-independent apoptosis process observed in SFN-treated and *Mabs*-infected cells. The three well-characterized MAPK subfamilies ERK, JNK, and p38 are involved in the pro- and anti-apoptotic pathways.^16^ PD98059, SB203580, and SP600125 are specific inhibitors of ERK, p38, and JNK, respectively, and were used to pretreat the macrophages before SFN pretreatment and/or *Mabs* infection. Inhibitory effect of PD98059, SB203580, and SP600125 was verified by immunoblotting using phosphorylated antibodies to ERK, p38, and JNK (data not shown). Interestingly, immunoblots showed that increased expression of Nrf2 in SFN-pretreated and *Mabs*-infected macrophages was not inhibited by the specific MAPK inhibitors suggesting that induction of Nrf2 expression is upstream from ERK, p38, and JNK pathways (Figure 7a).

To determine whether Nrf2 is implicated in the activation of ERK, p38, and JNK pathways, macrophages were transfected with siRNA designed to specifically silence Nrf2 expression levels 24 h before SFN and/or *Mabs* treatment. Western blotting of protein lysates extracted 24 h after infection showed a significant decrease in Nrf2 protein level although the abolition was not complete (Figure 7b).

In macrophages transfected with scrambled siRNA, SFN increased phosphorylation of ERK and p38 in SFN-pretreated macrophages and SFN-pretreated/*Mabs*-infected macrophages (Figure 7c). Phosphorylation of JNK was observed only in SFN-pretreated/*Mabs* macrophages. Infection with *Mabs* activated p38 with no significant effect on ERK and JNK. Interestingly, in macrophages transfected with siRNA targeting Nrf2, phosphorylation of ERK and JNK was higher in SFN-treated cells, *Mabs-*infected cells, and SFN-pretreated/*Mabs* cells compared with that seen in scrambled siRNA-transfected macrophages. These results suggest that SFN-induced Nrf2 has an inhibitory effect on ERK and JNK signaling pathways. Conversely, p38 phosphorylation is reduced to the basal level detected in DMSO-treated macrophages. Thus, Nrf2 controls the activation of the p38 pathway in our cell model (Figure 7c).

### p38 signaling pathway in the SFN/Mabs induced apoptosis

**Figure 8:**
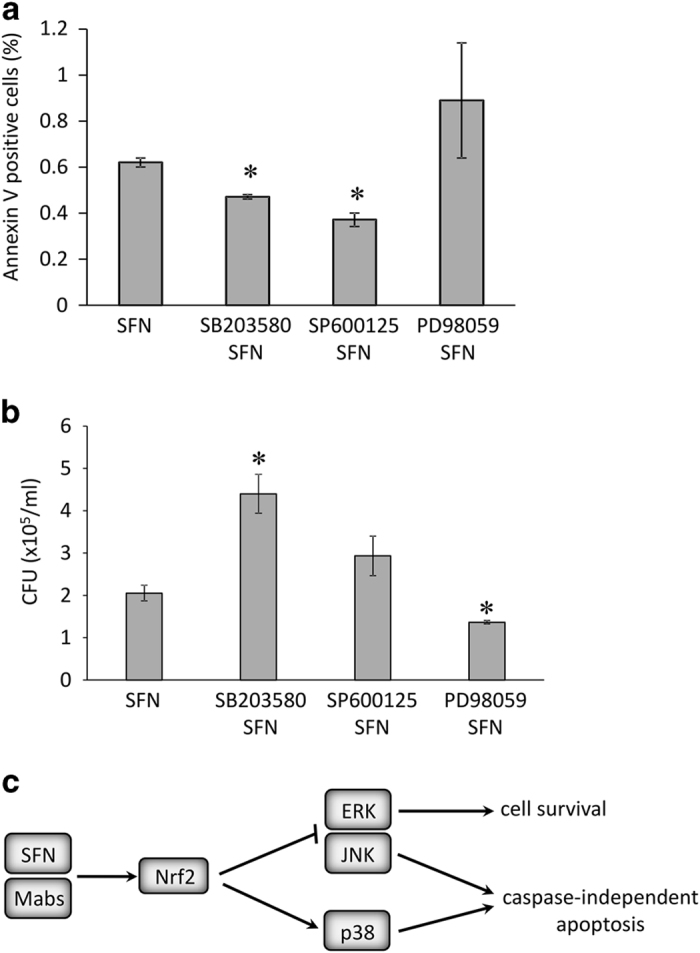
(**a**) Annexin V-FITC assay in THP-1-derived macrophages pretreated with MAPK inhibitors SB203580, SP600125, and PD98059. Infected cells were collected 24 h post infection. Data were from two independent experiments of *n*=10 000 events that were imaged and analyzed using MARKII imaging cytometer. *P<0.03 compared with SFN-treated cells. (**b**) THP-1-derived macrophages were pretreated with MAPK inhibitors before SFN pretreatment and *Mabs* infection. Mycobacteria were collected 48 h after infection by lysing cells in ice-cold water, serially diluted, and seeded on agar plates. CFUs were obtained 5 days after incubation at 37 °C. *P<0.03 compared with scramble transfected cells. (**c**) Caspase-independent cell death pathway activated by SFN and *M. abscessus* infection in THP-1-derived macrophages.

To determine whether p38 signaling pathway had a central role in the increase in cell apoptosis induced by SFN and *Mabs* infection, THP-1-derived macrophages were pretreated with MAPK inhibitors of p38, JNK, and ERK, before pretreatment with SFN and *Mabs* infection, and annexin V-FITC labeling was performed. A significant decrease was observed in apoptosis in SB203580- and SP600125-pretreated macrophages compared with SFN-pretreated cells, while no significant difference was observed in PD98059-pretreated cells (Figure 8a).

Moreover, THP-1-derived macrophages were pretreated with the MAPK inhibitors, SFN, and infected with *Mabs*. After 48 h, live mycobacteria were collected and seeded on LB-agar plates. The results showed a 2-fold increase in mycobacterial load in SB203580-pretreated cells, and a significant decrease in PD98059-pretreated cells (Figure 8b). Cells pretreated with SP600125 showed an increase in the mycobacterial burden that was not significant. The results validate further the implication of the pro-apoptotic p38 signaling pathway activated by Nrf2, and to a lesser extent JNK signaling pathway, in SFN/*Mabs*-induced apoptosis.

## Discussion

*Mabs* is able to cause skin, bone, and soft tissue infections and has been increasingly involved in exacerbations of lung infections and pulmonary diseases.^17^ Currently, its high resistance to antibiotics greatly limits patient treatment which may account for the likelihood of developing chronic airway infections and increase the risk of fatal outcome. Thus, development of new anti-mycobacterial drugs and treatment that may potentiate the actual drug therapies is urgently needed. SFN is a well-known activator of Nrf2 and has been shown to have several beneficial effects^18^ including an antibacterial effect on *H. pylori*.^19,20^ We used SFN as a pretreatment in our *in vitro* model of macrophage infection by *M. abscessus*. In this study, we describe a new mechanism by which SFN can inhibit bacterial proliferation. The important finding in this study is that SFN triggers a caspase-independent cell apoptosis in infected macrophages that requires activation of Nrf2 and p38 signaling pathways.

The role of oxidative stress in pathogen infection and propagation remains partially understood. Upon infection, microorganisms are detected, enveloped, and then phagocytosed by inflammatory cells from the innate immune defense system. These cells produce highly unstable and free radicals like ROS, comprising metabolites from partially reduced oxygen (superoxide anion, hydrogen peroxide, and hydroxyl radical), that will inflict irreversible damage to DNA, proteins, and lipids. This oxidative burst is crucial in pathogen clearance, but it appears that microorganisms, such as some mycobacteria, may survive and even preferentially thrive in an oxidative environment. *M. abscessus*, like *M. tuberculosis*, uses the host immune cells as a reservoir for proliferation and its growth is even enhanced in the presence of oxygen-free radicals.^5,21^ Here, we showed that although infection with *M. abscessus* induced ROS production in THP-1-derived macrophages, *M. abscessus* was able to prevent phagosomal pH acidification and thus proliferate intracellularly. Moreover, infection with *M. abscessus* led to an increase in cell necrosis with a negligible amount of cell apoptosis.

The consequence of this oxidative burst is an imbalance in oxidants/antioxidants, which activates triggering a cascade of cytoprotective and antioxidant defense mechanisms to maintain a redox homeostasis. This antioxidant cascade is controlled by Nrf2, the major transcriptional activator of ARE-mediated phase II enzymes. Although not fully understood, the Nrf2 signaling pathway seems to have an important role, either beneficial or detrimental, in microbial infections.^22^ Our results showed that infection of THP-1-derived macrophages with *M. abscessus* activates the antioxidant signaling pathway regulated by Nrf2. *M. abscessus* infection induced Nrf2 expression level and its translocation into the nucleus. Even though this activation is lower than that seen with SFN, it is still able to induce expression of HO-1 and NQO1, two downstream targets of Nrf2 (Figure 1). Recently, Abdalla *et al* ^23^ have described that infection of THP-1-induced macrophages by *M. abscessus* induced HO-1 expression and contributed to *M. abscessus* growth and survival in phagosomes of macrophages.^23^

Interestingly, *M. abscessus-*infected macrophages showed a significant decrease in mycobacterial growth 7 days post infection in cells pretreated with SFN compared with untreated cells, suggesting that activation of Nrf2 signaling pathway by SFN promotes mycobacterial growth inhibition. We showed that SFN had an effect neither in the early stage of pH acidification, nor on the phagocytosis mechanism since the amount of internalized *M. abscessus* was similar to infected macrophages. Interestingly, SFN showed no effect on either cell necrosis or cell apoptosis when macrophages were treated with SFN alone, but showed a significant increase in cell apoptosis in SFN-pretreated macrophages that were subsequently infected with *M. abscessus*. This apoptotic mechanism triggered by SFN in infected macrophages is in contradiction with the well-known protective effect of Nrf2 signaling pathway that promotes the survival of normal and cancerous cells.^24–26^

One efficient mechanism utilized by the innate immunity to fight mycobacterial infection is to undergo apoptosis. This mechanism has been reported to directly kill intracellular bacteria and apoptotic bodies enhance bacterial phagocytosis by activated and uninfected neighboring macrophages.^12^ One mechanism used by *M. tuberculosis* is to inhibit the apoptotic pathway to prevent the programmed death of infected macrophages and thus enhance its intracellular survival.^27,28^ Here, Nrf2 and HO-1 protein expression levels were strongly induced in SFN-pretreated and *M. abscessus-*infected macrophages and this observation correlates with an increase in cell apoptosis in these cells. Macrophages pretreated with SFN alone or infected with *M. abscessus* alone did not have any apoptotic effect. We demonstrated that cell apoptosis induced by SFN pretreatment in infected macrophages was caspase independent and p38 MAPK dependent (Figure 8c). It is likely that, as for *M. tuberculosis*, *M. abscessus* is also able to inhibit the typical apoptosis pathway in the infected macrophages causing the cells to induce the caspase-independent apoptosis that we observe in our study.

Various caspase-independent cell death have been recently described in the literature. The cell death mechanistic pathway triggered by SFN in infected macrophages strongly resemble the defense mechanism activated by the innate immunity following viral infection and previously described as necroptosis.^29,30^ This alternate cell death pathway is triggered in phagocytes to help eliminate phagocytosed viruses that are able to block the classical apoptosis pathway. We speculate that SFN may be able to help *M. abscessus*-infected macrophages overcome the blockade in cell apoptosis and eliminate mycobacteria by triggering a mechanism similar to necroptosis. One other caspase 3-independent mechanism that has been recently implicated in *M. tuberculosis*-infected macrophages is the inflammation-related pyropotosis.^31^

In conclusion, the present study showed that activation of the Nrf2 signaling pathway by SFN can reduce *M. abscessus* proliferation in macrophages by inducing a caspase-independent cell apoptosis. To our knowledge, we describe for the first time the involvement of Nrf2 and p38 signaling pathways in the mechanism activated by sulforaphane and involved in inhibition of bacterial proliferation. These findings indicate that modulation of the Nrf2 signaling using Nrf2 activators may potentiate the actual multi-drug therapies used to treat patients diagnosed with mycobacterial infection.

## Materials and Methods

### Antibodies and reagents

SFN, PMA, and amikacin were purchased from Sigma-Aldrich (Saint-Quentin Fallavier, France). RPMI-1640, penicillin/streptomycin, HEPES, sodium pyruvate, fetal bovine serum (FBS), fluorescein-5-isothiocyanate, LysoTracker Red DND-99, and pre-designed Nrf2 siRNA (Ambion, Fisher Scientific, Illkirch, France), Middlebrook 7H9 broth and Oleic acid/bovine albumin/dextrose/catalase enrichment (Difco, Becton Dickinson, Le Pont de Claix, France), and ECL prime detection reagent (GE Healthcare, Buckinghamshire, UK) were purchased from Fisher Scientific. HiPerfect transfection reagent and scrambled siRNA were purchased from Qiagen (Courtaboeuf, France). DC protein assay kit was purchased from Bio-Rad (Marne-la-Coquette, France). Complete protease inhibitor cocktail tablets were purchased from Roche (Mannheim, Germany). 2ʹ,7ʹ- dichlorodihydrofluorescein diacetate (Calbiochem, San Diego, CA, USA), anti-phospho ERK1/2, and anti-MAPK were purchased from Millipore (Nottingham, UK). MAPK inhibitors PD98059, SB203580, SP600125, annexin-V FITC apoptosis detection kit, anti-lamin A/C, anti-*β* actin, and anti-HO-1 antibodies were purchased from Abcam (Cambridge, UK). Anti-phospho p38, anti-p38, anti-phospho JNK, and anti-JNK were purchased from BD Biosciences (Le Pont de Claix, France). EurobioGreen qPCR mix and FAM-FLICA Caspase 3/7 detection kit (ImmunoChemistry Technologies, Bloomington, MN, USA) were purchased from Eurobio (Courtaboeuf, France). Anti-Nrf2 antibody (Santa Cruz, Dallas, TX, USA) and Fluoromount-G (Southern Biotech, Birmingham, AL, USA) was purchased from Clinisciences (Nanterre, France). HRP-conjugated anti-mouse or rabbit IgG were purchased from Jackson ImmunoResearch laboratories (Suffolk, UK).

### Cell culture

The human THP-1 monocytic cell line was maintained in RPMI-1640 medium with Glutamax supplemented with 10% heat inactivated FBS, 1 mM sodium pyruvate, 10 mM HEPES, 1% penicillin-streptomycin, and 0.05 mM *β*-mercaptoethanol in a humidified atmosphere at 37 °C and 5% CO_2_. Cells were maintained at a density between 2.5×10^5^ cells/ml and 1×10^6^ cells/ml. Terminal differentiation of THP-1 to macrophages was obtained by rinsing the cells twice with PBS before treatment with 10 *μ*M PMA for 48 h. Depending on the indicated conditions, THP-1-derived macrophages were pretreated with 10 *μ*M sulforaphane or DMSO for 3 h before mycobacterial infection. When indicated, cells were pretreated with MAPK inhibitor (PD98059, SB203580, or SP600125) 1 h before SFN pretreatment and/or *M. abscessus* infection.

### Mycobacterial strain

*M. abscessus* strain used for this study displayed a smooth morphology. *Mabs* expressing mCherry or GFP fluorochrome was generated by transforming *Mabs* with mCherry or GFP expressed pMV261-kanamycin plasmid. *Mabs*-mCherry and *Mabs*-GFP were cultured aerobically at 37 °C in Middlebrook 7H9 broth supplemented with 0.05% Tween-80, 10% (v/v) oleic acid/albumin/dextrose/catalase enrichment, and supplemented with the appropriate antibiotics (250 *μ*g/ml kanamycin and 1 mg/ml hygromycin, respectively). Before infection, bacteria were washed twice in PBS and single bacilli were obtained by passing the bacteria suspension sequentially through a 25-G needle and a 29-G insulin syringe 10 times each. The number of bacteria was determined by counting the fluorescent bacteria in a Malassez counting chamber using an epifluorescence microscope.

### *In vitro* cell infection and intracellular growth measurements

Forty-eight hours after seeding THP-1 in 24-well plates at 5×10^5^ cells/well, and inducing differentiation to macrophages with PMA, the cells were infected with *Mabs*-mCherry at the MOI of 10:1 (bacilli to THP-1). After 3 h infection at 37 °C and 5% CO_2_, unincorporated bacilli were eliminated by thoroughly washing twice with PBS. Infected and control cells were then treated for 1 h with 250 *μ*g/ml amikacin to eliminate the remaining unattached bacilli, washed with PBS, and cultured for up to 7 days in culture cell medium supplemented with 50 *μ*g/ml amikacin. The CFU counts were determined at day 0, 1, 3, and 7 after infection. The intracellular bacilli were collected by lysing cells with ice-cold distilled water and plating 10-fold serial dilutions on Luria Bertani agar plates. The number of bacilli was determined by counting individual colonies after 5–7 days of growth at 37 °C.

### Phagosomal acidification assay

*Mabs*-mCherry was surface labeled with the pH-sensitive FITC. THP-1 seeded in 24-well plate at 5×10^5^ cells/well was infected with doubly labeled *Mabs* at the MOI of 10:1 for 20 min at 4 °C. After washing with PBS, and supplementing the cells with PBS-1% FBS, fluorescence signal intensities were detected using the Fluoroskan Ascent FL spectrophotometer (Fisher Scientific). FITC and mCherry signal intensities were acquired every 5 min for 1 h then every 10 min for 2 h at 34 °C by sequential excitation at 485 and 544 nm, respectively. For each experiment, a standard pH curve was determined by correlating the fluorescence intensities to standardized pH buffers.

### Cell transfection

To knockdown Nrf2, THP-1-derived macrophages were transfected 24 h before pretreatment or infection with a pre-designed Nrf2 siRNA (siNrf2), or a universal control RNAi (scramble), using HiPerfect transfection reagent following the manufacturer’s recommended protocol.

### Subcellular fractionation and immunoblotting

For immunoblot analysis, THP-1-derived macrophages were rinsed with cold PBS then lysed with cold RIPA buffer (150 mM NaCl, 1% Triton X-100, 0.5% sodium deoxycholate, 0.1% SDS, 50 mM Tris-HCl, pH 7.5, supplemented with Complete protease inhibitor cocktail mixture). Protein concentrations were determined using DC protein assay kit. Twenty micrograms of total proteins were resolved by SDS-PAGE (4–20% gradient gels) and transferred onto polyvinylidene difluoride membrane. Membrane blocking was performed in 5% BSA/TBST (10 mM Tris-HCl, pH 7.4, 150 mM NaCl, and 0.1% Tween-20) for 1 h before incubation with primary antibodies. The corresponding horseradish peroxidase-conjugated secondary antibodies were used at dilution 1/20 000. Immunoreactivity was visualized using the ECL prime detection reagent and detected using the QuantityOne software (ChemiDoc XRS, Bio-Rad, Marne-la-Coquette, France). Immunoblots shown are representative of three independent experiments.

### Total RNA isolation, reverse transcription, and quantitative real-time PCR

Total RNA was isolated from treated cells using the RNeasy Mini kit (Qiagen). RNA concentration and purity were determined using the GE NanoVue spectrophotometer (GE Healthcare). RNA was reverse transcribed into cDNA using Superscript III First strand synthesis kit (Life Technologies, St Aubin, France) with an oligo(dT) primer, according to the manufacturer’s instructions. cDNA was analyzed using real-time qPCR, with each sample done in triplicate. qPCR was performed using the CFX96 thermocycler (Bio-Rad) and EurobioGreen qPCR mix. The specific oligonucleotides for Nrf2, HO-1, and NQO1 were designed using Primer-BLAST (NCBI website), and purchased from Eurogentec (Angers, France; Supplementary Table 1). Gene expression levels were calculated as a ratio to the expression of the reference gene ubiquitin C (UBC). Data were analyzed on the Bio-Rad CFX manager 3.1 using the ΔΔCt method.

### Apoptosis and necrosis assays, activated Caspase 3/7 assay, and ROS production assay

Apoptosis assay was performed according to the manufacturer’s procedure. Briefly, THP-1 cells cultured in 6-well plate at 1×10^6^ cells/well were pretreated with SFN or DMSO for 3 h before mycobacterial infection. At the indicated times, cells were trypsinized, washed once with PBS, resuspended in Annexin binding buffer 1×, and stained with Annexin V-FITC for 5 min in the dark and at room temperature. After one PBS wash, the cell pellet was suspended in 100 *μ*l Annexin binding buffer 1×. Quantification of necrotic THP-1-derived macrophages was performed by incubating the infected and non-infected cells with 2 *μ*g/ml PI.

For assessing Caspase 3/7 activity in THP-1 cells, the non-cytotoxic FLICA probe was used. The FLICA probe, composed of the irreversible caspase inhibitor DEVD-fluoromethyl ketone fused to a carboxyfluorescein, binds specifically and covalently to activated Caspase 3/7 enzymes. Following the manufacturer’s protocol, cells were incubated 1 h at room temperature and in the dark. After two washes, the cells were resuspended in wash buffer 1×.

The generation of ROS in THP-1-derived macrophages was monitored using the cell-permeable fluorogenic probe, 2’,7’-dichlorodihydrofluorescein diacetate (H_2_DCFDA). Treated cells tested for apoptosis, necrosis, ROS generation, and activated caspases 3/7 production were analyzed by imaging flow cytometry (MARK II, Merck-Millipore, Nottingham, UK) as described below.

### Imaging flow cytometry

Apoptotic/necrotic/infected cells, H_2_DCFDA-labeled cells, and activated Caspase 3/7 FLICA-labeled cells were quantified using ImageStream Mark II (Amnis, Merck-Millipore) imaging flow cytometer, which allows simultaneous imaging and analysis of cells. Depending on the assay, data from 5000 to 10 000 events were acquired at ×40 magnification and using 488, 658, and 785 nm lasers. Compensation settings were adjusted on single-color controls for each fluorochrome and analyses were performed using the IDEAS 5.0 data analysis software (Amnis). The brightfield images were used to verify cell integrity. A brightfield area *versus* brightfield aspect ratio scatterplot was used to gate on single cells and eliminate cell aggregates. The single cells were then plotted on an Annexin V bright detail intensity *versus* brightfield area to gate on the Annexin V^+^ cells. The Annexin V^+^ cells were then plotted on an *M. abscessus*-mCherry bright detail intensity *versus Mabs*-mCherry area to gate on the Annexin V^+^ *Mabs* ^+^ cells. The same procedure was applied to the PI, FLICA, and H_2_DCFDA labeling.

### Lysosomal labeling and indirect immunofluorescence imaging

THP-1-derived macrophages were seeded at 2.5×10^5^ cells per well on coverslips in 24-well plates and were incubated in PMA-containing medium. After 48 h incubation, cells were transfected using Hiperfect transfection reagent (Qiagen) and scrambled or siNrf2 24 h before pretreatment with MAPK inhibitor 4 h before infection. SFN or DMSO pretreatment was done 3 h before infection with *Mabs*-GFP for 3 h. Labeling with LysoTracker Red DND-99 (Life Technologies) was done according to the manufacturer’s instructions. Cells were rinsed with PBS and fixed with 4% paraformaldehyde at room temperature for 30 min. After one rinse with PBS, coverslips were mounted on slides using Fluoromount-G. Cells were observed using a confocal microscope (Leica TCS SPE, Nanterre, France). Images were treated and analyzed using ImageJ software (National Institutes of Health, Bethesda, MD, USA).

### Statistical analyses

Results are presented as means±S.E.M. of three independent experiments done in triplicates. Imaging flow cytometry results presented are means±S.E.M. of two to three independent experiments of 5000–10 000 events. Statistical comparisons were performed using two-tailed Student’s *t*-test and differences were considered to be significant at a value of *P*<0.05.

## Abbreviations

CFU: colony-forming unit
DMSO: dimethyl sulfoxide
ERK: extracellular signal-regulated kinase
FITC: fluorescein isothiocyanate
H_2_DCFDA: 2',7'-dichlorodihydrofluorescein diacetate
HO-1: heme oxygenase-1
JNK: c-Jun N-terminal kinase
Mabs: *M. abscessus*
MAPK: mitogen-activated protein kinases
NQO1: NADPH quinone oxidoreductase-1
Nrf2: Nuclear factor E2-related factor 2
PCR: polymerase chaine reaction
RGM: rapid growing mycobacteria
ROS: reactive oxygen species
SFN: sulforaphane
siRNA: small interfering RNA
