# Supplementary material for: Caspase-independent apoptosis in infected macrophages triggered by sulforaphane via Nrf2/p38 signaling pathways
Source: Cell Death Discov. 2015 Aug 24;1:15022–. doi: 10.1038/cddiscovery.2015.22 (PMC4979433; doi:10.1038/cddiscovery.2015.22)
Supplement: Supplementary Table 1 [file cddiscovery201522-s1.pdf]

Gene	qPCR primer sequences (5’ $\rightarrow$ 3’)
Human Nrf2-F	AGCGACGGAAAGAGTATGAG
Human Nrf2-R	GTTGGCAGATCCACTGGTTT
Human HO-1-F	TCCGATGGGTCCTTACACTC
Human HO-1-R	TAAGGAAGCCAGCCAAGAGA
Human NQO-1-F	CAGACGCCCGAATTCAAATC
Human NQO-1-R	AGGCTGCTTGGAGCAAAATACA
Human UBC-F	CACTTGGTCCTGCGCTTGA
Human UBC-R	TTTTTTGGGAATGCAACAACTTT

Supplemental Table 1
